# Education Based on Precede-Proceed on Quality of Life in Elderly

**DOI:** 10.5539/gjhs.v6n6p178

**Published:** 2014-07-29

**Authors:** Saeed Mazloomymahmoodabad, Gholamreza Masoudy, Hosain Fallahzadeh, Zahra Jalili

**Affiliations:** 1 Department of Health Education and Promotion, Shahid Sadoughi University of Medical Sciences, Yazd, Iran; 2 Health Research Center, Zahedan University of Medical Sciences, Zahedan, Iran; 3 Department of Biostatistics, Shahid Sadoughi University of Medical Sciences, Yazd, IR Iran; 4 School of Medical Sciences, Islamic Azad University (I.A.U), Tehran, Iran

**Keywords:** education, elderly, precede- proceed, Quality of Life

## Abstract

**Background and Objective:**

One of the most important challenges in public health is to improve the quality of life in elders. Aging may cause various disorders such as disabilities, high risk conditions and some chronic disease. In this study the effect of educational intervention based on precede–proceed on quality of life in elders was examined.

**Materials & Methods:**

This semi experimental study was carried out on 128 elders over 60 years in Zahedan that were randomly selected by multi-stage sampling method and divided in to control and intervention groups. Data collection tool was a triploid questionnaire that included demographic data, questions of precede-proceed constructs and SF-36 questionnaire. The validity and reliability of questionnaire confirmed by experts and Cranach’s Alpha coefficient (76%). After primary data collecting, educational intervention was performed and after nine months data was collected again and analyzed in spss.16 soft-ware using descriptive and analytical statistics.

**Results:**

The results showed that mean score of quality of life in participants was low and more than 61% of them had a mean score less than 50%. After intervention the mean score of quality of life only in experimental group significantly increased from 47.72 to 58.90. Behavior and self-rated health were the strongest predictors for quality of life in this study.

**Conclusion:**

Implementation educational intervention based on precedes-proceed model can improve quality of life in elders. Elderly women and older elderly individuals compared with elderly men and younger elderly should be considering as an important risk factor for reducing HRQOL.

## 1. Introduction

Aging is an ancient subject and a sensitive period in human life and a normal, biological, universal process and unavoidable phenomenon, (Mudey et al., 2011; Qadri, Ahluwalia, Ganai, Bali, Wani, & Bashir, 2013; Wu, 2010) From 1965 to 2025, the percentage of elderly persons (aged ≥ 65 years) is expected to increase by 17 to 82% in European countries and by about 200% in some developing countries (Aghamolaei, Tavafian, & Zare, 2010).

Similar to most other countries, as a result of Improvement in public health and medical advances life expectancy in Iran has increased over the last decades such that it has been measured as 70.3 years in 2005 while it has been 47.2 years in 1960, 52.5 years in 1970, 57.7 years in 1980, 63 years in 19 90 and 68.6 years in 2000 (Aghamolaei et al., 2010).

Population aging has many health consequences, including increase in the old-age dependency ratio and disability. Elders have tendency for poorer health status compare younger and population aging cause concerns about potential increases in the number of elderly that suffering of disease and disability (Annear, Cushman, & Gidlow, 2009).

Finding showed that the psychosocial and social disorders decrease the level of health and quality of life in elderly and has an association with death in elderly (Huong, Hai, Quynh, Hill, & Walton, 2012; Kumar & Majumdar, 2014; Qiua, Sautterb, Liuc, & Gud, 2011; Whatley, DiIorio, & Yeager, 2010). As life expectancy continues rising; one of the greatest challenges of public health is to improve the quality of life in later years of life.

The progressive rise in life expectancy contributes to an increase in the prevalence of chronic illnesses such as Muscular pain, joint pain, restlessness, headache, visual problems, and high blood pressure, cardiovascular disease, digestion and stomach problems, diabetes in the elderly population (Zhou, C. Wang, Yang, Z. Wang, Zheng, & J. Wang, 2014).These disorders and many other health problems are result of poor health and unsanitary practices

The concept of quality of life contains satisfaction and wellbeing, comprising subjective and multi-dimensional characteristics. Quality of life can be addressed as general quality of life or health-related quality of life (HRQOL) (Lima et al., 2009).

One of the most widely used instruments to assess health-related quality of life is the SF-36 questionnaire (Medical Outcomes Study 36-item Short-Form Health Survey) that is drawn of the Medical Outcomes Study (MOS) questionnaire. SF-36 questionnaire published in English in 1990 (Whatley et al., 2010).

SF-36 translated and validated in several languages and cultures such as Persian and applying in more than 40 countries (Lima et al., 2009; Montazeri, Goshtasebi, Vahdaninia, & Gandek, 2005).

Each of the sf-36 questionnaire questions refers to one of the following eight different health indicators: physical functioning, role-physical and bodily pain that they refer to the general factor of physical health.

Scales social functioning, role-emotional and mental health measure psychological health and Scales vitality and general health are moderately connected with both factors (Amela et al., 2012; Burholt & Nash, 2012).

The SF-36 Manual recommended that norm-based scores (NBS) should be used rather than 0-100 scores to simplify interpretation of the data (Burholt & Nash, 2012).

The elderly in Iran like other developing societies are facing many health and social challenges such as: illiteracy, economic difficulties, problems with daily living, life dissatisfaction, lack of medical insurance, as well as mental and emotional problems (Tajvar, Areab, & Montazeri, 2008).

In general in health promotion programs, applying models is suggested because models explain how behaviors occur, how health education is applied and how this education affects latter behaviors (Sharifirad, Ghaffari, Zanjani, & Hassanzadeh., 2012).

Precede-proceed model is a planning model and the best available framework to describe factors influencing health outcomes and provide a comprehensive structure for health needs assessment, program design, implementation and evaluation of health promotion programs, behavior studying, and health promotion (Ekhtiari, Shojaeizadeh, Rahimi Foroushani, Ghofranipour, & Ahmadi, 2013; Phillips, Rollexy, & Davidson, 2012).

The PROCEED phases include social, epidemiological, behavioral and environmental, educational and ecological, administrative and policy assessment (Phillips et al., 2012).

Based on precede-proceed model Assumptions and many other finding, predisposing, reinforcing and enabling factors have an effect on behaviors and quality of life (Khaldoun, Aldiabat, & Navenec, 2013).

Zahedan is one of the southern cities in Iran that located in Sistan and Baloochestan province. Comparison of quality of life mean scores in Zahedan and other cities showed that the mean scores of quality of life of elderly in Zahedan were lower than other cities (Nejati, 2008).

## 2. Material and Method

The sampling method was multi-stage Sampling. In the first stag based on socio economic condition all health centers divided in to 5 sections and then randomly selected a health center of every category. In the next stage from every five health centers, randomly two regions for control and experimental groups were selected.

To obtain the participants based on population ratio in every health center, subjects were selected randomly for both groups (control and intervention). Generally 64 participants for each group selected and surveyed. Data collection tool was a triploid questionnaire included SF-36 questionnaire, demographic items and questions based on precede-proceed model such as knowledge and attitude questions (predisposing factors), reinforcing factors, Behavioral and enabling factors questions.

Regarding the results of a study that showed educational intervention based on elderly family members had a significant impact on quality of life in elderly (Rabiei, Mostafavy, & Masoudi, 2013), we developed an educational booklet for elder’s family that they should study it and support the educational program. Family members were asked to help elderly in accomplishing some healthy behaviors such as physical activity, following proper nutrition and medicine adherence.

Besides usual constructs of precede-proceed we added a further construct as environmental construct that included 14 questions about health condition of elderly housing for measuring the physical environment status of participants in this study.

The validity and reliability of questionnaire was approved by content validity method and Cronbach’s Alpha coefficient. The validity of questionnaire was confirmed by 10 relevant experts. Cronbach’s Alpha coefficient for reliability was 76 percent.

Also we used SF-36 questionnaire for measuring the quality of life and a self-rated health questionnaire to measure the health status of participants. For data collection at first the research objectives were explained and then primary information in both groups were collected and analyzed by SPSS16. Then educational content and curriculum were adjusted based on these. Educational media was a booklet and a video that prepared in two languages [(Persian and baloochy (dialect)].

## 3. Results

A total of 128 individual’s age over 60 years participated in this study. The mean age of the participants in the case and control group was 65.80 and 67.80 years, respectively. It was found that 48.4 and 46.6 percent of the participants in the interventional and control group were illiterate. In addition, 82.8% of the elderly in the case group and 89.1% of them in control group lived with their spouses and their single children. Also 17.2% and 21.9% of participants in the case and control groups had a history of broken bones in their medical history in the past year.

The results showed that no significant difference was seen between two groups before of intervention stage in terms of the level of the education, sex and current source income. Also results from independent t- test before intervention showed that no significant difference was seen between two groups in mean score of the precede- proceed constructs [Knowledge and attitude (predisposing factors), enabling factors, reinforcing factors and behavior] environment conditions, self-rate health and quality of life (p > 0.05) but after the intervention a significant difference was seen (p < 0.05) (Table 1).

**Table 1:** mean scores of knowledge, attitude, behavior, enabling factors, reinforcing factors, environment- conditions, self-rate health and quality of life in Case and control group in before and after intervention

Variable	Before			After		

	Case M (SD)	Control M (SD)	p- value	case M (SD)	Control M (SD)	p- value
knowledge	20.609(3.16)	21.187(3.67)	0.342	24.54 (3.54)	19.406(4.67)	0.001
Attitude	25.6250 (3.15)	25.562(2.79)	0.906	27.48 (2.05)	25.421(2.83)	0.001
Behavior	14.953(3.76)	14.875(3.78)	0.907	18.062 (3.7)	15.218(4.142)	0.001
Enabling factors	19.053(2.39)	19.765(2.38)	0.658	21.938 (1.94)	19.812(2.31)	0.001
Reins factor	21.953(1.41)	21.546(2.22)	0.220	23.078(1.34)	21.187(2.15)	0.001
Environment conditions	19.593(1.91)	19.078(1.67)	0.108	21.0938(2.15)	19.031(1.69)	0.001
Self-rated health	7.390(2.15)	6.921(2.39)	0.247	8.671(2.21)	6.609(2.29)	0.001

Data are reported as mean (SD)

Data are reported as mean (SD)

Also paired T- Test results showed that after the intervention only in case group, the mean scores of the precede –proceed constructs, self-health rated and quality of life significantly increased (p < 0.05).

Before intervention, the mean scores of quality of life were 47.72 and 44.73 in the case and control groups, respectively. Before intervention no significant difference was seen between two groups in mean score of SF-36 scales (p > 0.05), but after intervention a significant difference between two groups was seen (p < 0.05) (Table 2).

**Table 2:** Mean scores of SF-36 Scales in Experimental and control group in before and after of intervention

Scale	Before			After		

	case M (SD)	control M (SD)	P value	Experimental M (SD)	control M (SD)	P value
Physical functioning	38.59(24.93)	39.84(27.00)	0.76	50.31(27.03)	38.51(24.71)	0.001
Role-physical	38.28 (33.62)	37.10 (34.50)	0.846	56.25 (34.21)	34.37(34.06)	0.001
Bodily pain	41.56 (25.27)	39.37 (27.93)	0.643	52.50 (25.94)	37.34(28.68)	0.002
General health	58.59 (18.11)	54.29 (17.04)	0.169	65.39 (19.98)	51.17(18.16)	0.001
Vitality	54.68 (22.340	49.21 (24.54)	0.190	60.93 (20.15)	49.68(23.1)	0.004
Role-emotional	38.02 (38.42)	36.45 (35.49)	0.812	60.93 (35.90)	32.29(33.58)	0.001
Social functioning	46.67 (23.80)	43.55 (24.8)	0.468	54.8 (22.004)	43.16(23.66)	0.004
Mental health	65.37 (20.08)	58.06 (22.88)	0.057	70.00 (20.18)	57.37(21.53)	0.001
Quality of life	47.724 (17.82)	44.739 (18.92)	0.360	58.90 (18.57)	42.990(18.75)	P<0.00

Data are reported as mean (SD).

In control group Paired T-Test results showed that no significant difference was seen in mean scores of SF-36 scales group in before and after intervention stages (p > 0.05), but after intervention the mean scores of SF-36 scales in case group were increased significantly (p < 0.05).

Pearson’s correlation coefficients (r) indicated that quality of life was significantly correlated with knowledge (r = 0.723, p < .001), attitude (r = 0.311, p < 0.001), behavior (r = 0.665, p <.001), enabling factors (r = 0.333, p < .001), reinforcing factors (r = 0.440, p < .001), environmental condition (r = 0.300, p < .001), and self- related health (r = 0.723, p <.001).

A regression model including behavior (β = 0.381, Standard Error = 0.320, p = 0.001) and self-health related (β = 0.510, Standard Error = 0.540 p = 0.001) was significantly predict the quality of life that explained 0.623 of QOL variances.

## 4. Discussion

The comparison of quality of life of elderly in Zahedan and in some other cities in Iran showed that they had lower mean scores for quality of life and all of the SF-36 health survey scales. This important finding properly suggests that health promotion programs should be considered and implemented (Lima et al., 2009).

Regarding the results of this study, educational intervention based on precedes- proceed increased all of mean scores of precede-proceed, SF-36 health survey scales, and mean score of quality of life in the elderly. Sharifirad et al found similar results after the educational intervention; the case group got higher scores in predisposing (knowledge and attitude), enabling, and reinforcing factors (Sharifirad, Ghaffari, & Hassanzadeh, 2012).

The present study showed that the mean score of quality of life in elderlies that participated in this study was low and more than 61% of them had a mean score less than of 50. Also the present study showed that women compare to men had a lower mean score in quality of life in before and after intervention stage and this results are supported by results of other researches in Iran and other countries which showed that women, old people and low income elderly had poorer health compare men and the younger people. (Aghamolaei1 et al., 2011; Franzen, Saveman & Blomqvist, 2007; Guallar et al., 2005; Francesc et al., 2006).

Regarding this study women and older elderly have more risk factor for reducing HRQOL, and health educational designers, policymakers and health planners should pay more attention to them for designing the more effective interventions to prevent the effects of aging on HRQOL.

The results of independent T-test in before intervention indicated that no significance difference were seen between two groups in mean scores of knowledge, attitude, enabling factors, reinforcing, environment, behavior and self-related health but after intervention there were significant difference among them.

The results of present study in after educational stage showed that the mean score of quality of life in control group unlike experimental group reduced. It is an important finding because indicated aging and environmental conditional influence the elderly, and there is a necessary for health promotion interventions. This result is supported by some article in Iran or other countries (Thumboo et al., 2003; Chao et al., 2012; Orfila, Ferrer, Lamarca, Tebe, Domingo-Salvany, & Alonso, 2006).

According to result of linear regression analysis, behavior and self-rated health were the strongest predictors for quality of life, and comparison of this variable in men and women indicated that women have a lower mean score in quality of life.

So we conclude that in health promotion programs designing should pay more attention to women and behavior change programs should be considered and visual media is suggested.

## 5. Conclusion

This study showed that after education intervention based on precede-proceed model the case group got higher scores in knowledge, attitude, behavior, reinforcing and quality of life compare to the control group. So we can conclude that the precede-proceed model provided an applicable framework for apply educational program in elderly.

The analysis of results in present study showed that women compared to men in before and after educational intervention gut lower mean scores in quality of life; therefore it is necessary that in health interventions for promoting healthy behaviors in elders should pay more attention to women.

The results of this study showed that using training videos or booklet that had adequately simple educational pictures were proper strategies to increase of knowledge of elderly.

Also with regard to role of cultural differences, especially different language as a communication barrier, it is necessary that in designing educational program for achieving better results, public health planners should pay more attention to language and cultural conditions of participants.
